# Team sport expertise shows superior stimulus-driven visual attention and motor inhibition

**DOI:** 10.1371/journal.pone.0217056

**Published:** 2019-05-15

**Authors:** Fan-Wu Meng, Zai-Fu Yao, Erik Chihhung Chang, Yi-Liang Chen

**Affiliations:** 1 Graduate Institute of Sports Training, University of Taipei, Taipei, Taiwan; 2 Office of Physical Education, Chung Yuan Christian University, Taoyuan City, Taiwan; 3 Brain and Cognition, Department of Psychology, University of Amsterdam, Amsterdam, The Netherlands; 4 Institute of Cognitive Neuroscience, National Central University, Taoyuan City, Taiwan; University of Birmingham, UNITED KINGDOM

## Abstract

Previous studies on athletes’ cognitive functions have reported superior performance on tasks measuring attention and sensorimotor abilities. However, how types of sports training shapes cognitive profile remains to be further explored. In this study, we recruited elite athletes specialized in badminton (N = 35, female = 12) and volleyball (N = 29, female = 13), as well as healthy adult controls (N = 27, female = 17) who had not receive any regular sports training. All participants completed cognitive assessments on spatial attention, sensory memory, cognitive flexibility, motor inhibition, and the attention networks. The results showed that athletes generally showed superior performance on selective cognitive domains compared to healthy controls. Specifically, compared to the healthy control, volleyball players showed superior on iconic memory, inhibitory control of action, and attentional alerting, whereas badminton players showed advantages on iconic memory and basic processing speed. Overall, volleyball players outperformed badminton players on those tasks require stimulus-driven visual attention and motor inhibition, likely due to different training modalities and characteristics of specialty that involves even more complex cognitive processes. To conclude, our findings suggest cognitive plasticity may drive by sports training in team/individual sports expertise, manifesting cognitive profile in sport expertise with distinct training modalities.

## Introduction

Recent years have seen accumulating evidence that physical activity and cardiovascular fitness are positively associated with cognitive functions [1–3]. Structural and functional brain changes have also been linked to this “fitness effects” of physical exercise [4,5]. Although several studies have shown the benefits of physical training lasting from a few tens of minutes to months on cognitive functions, little is known about how very long-term physical training shapes cognitive profiles, such as athletes who have devoted tremendous time and efforts in their specialty since very young in age.

A recent study have highlighted the missing link in the field of sport expertise and its brain function [6], that is, whether sports expertise with decades of physical training would lead to superior performance on general or specific cognitive skill. A meta-review concluded that elite athletes had better performance on visual attention and basic sensorimotor processing speed, especially those who are specialized in interceptive sports [7]. In their review study, Voss et al. distinguishes three sports types: static, interceptive, and strategic sport. The *static* sports involve highly consistent and self-paced situations (e.g. running and swimming); the interceptive sports require coordination of varied parts of body or a held implement to catch or reach a pass by object in the dynamic environment (e.g. tennis, fencing, and boxing); the strategic sports require concurrent processing of substantial amount of information such as teammates, opponents, field position and ball, and often involve highly varied situations (e.g. volleyball, basketball, soccer, hockey, field-hockey, and water-polo). Many studies have since emerged to investigate the relationship between sports expertise and different aspect of cognitive functions. For example, tennis players (i.e., interceptive sports) showed better performance on motor inhibition [8] and sensorimotor speed [9], while soccer players have superior performance on visual attention [10] and executive functions [11–13] than controls. The results seem to converge on the view that athletes have better performance on the task measuring executive functions[11,14] and varied attention abilities [15–17]. Nonetheless, sports-related superior cognitive performance is not only observed when dealing with stressful situation [18] but also predictive of actual game performance [19,20].

Although quite a few studies have demonstrated that athletes appear superior to lay person at certain aspect of cognitive abilities, the findings across various cognitive tasks assessing athletes with different types of sports expertise are still mixed [8,16,21,22]. For example, while a recent study demonstrated that team sports athletes performed better in a basic cognitive test of sustained attention and processing speed than recreational athletes [16], opposite conclusions have also been made in a study comparing basketball, volleyball and water-polo players on a battery of cognitive tests, which found that not all types of experts outperformed novices on certain type of tasks of the battery [23]. Moreover, Chang and colleagues [21] examined the differences between types of sports expertise (i.e., endurance athletes versus Wushu martial art athletes) and non-athlete controls on general cognition. Despite both athletes group did show higher levels of cardiovascular and motor fitness than the control group, no significant difference was found between the three groups for the battery of general cognitive tasks.

The current study aims to explore how cognitive profiles differ among types of sports expertise. We hypothesize that sports expertise experience from different training modalities would manifest itself in distinct cognitive skills [7]. We contrasted two types of athletes that have been shown to excel in cognitive functions in general, namely those who specialized in interceptive sports (i.e., badminton player) and strategic sports (i.e., volleyball player). Strategic sport players have to coordinate their skills with multiple players on-the-fly, leading to the most loading of information processing demand from their coach, teammates, and opponents. The loading can involve executing tactics, updating the location of teammates/opponents, and following the rules during the game. The interceptive sports players have to interact with their opponents with an instrument and need to move at extremely high speed, such as reaching to catch a ball or running towards a target to make a tackle. This implies precise control of intended action [8], proper reaction to target [24], and locomotion in optimal combinations of space and time [18,25–27]. Based on the characteristics of strategic sports [28] and interceptive sports [24], we hypothesized that strategic-sports players should exhibit better performance on task measuring attention and executive functions, whereas interceptive-sports players should show better performance on motor inhibition and basic processing speed.

## Materials and methods

### Participants

There were 91 participants, including 35 elite badminton players (mean age 22.7±3.4 years, female = 12), 25 volleyball players (mean age 23.6±2.8 years, female = 13), and 27 healthy non-athletes control (mean age 22.8±3.2 years, female = 17). The Badminton players were all professional athletes who had represented Taiwan in international competitions. Sixteen of them ranked among the top 100 in the Badminton World Federation Ranking. Young volleyball players were recruited from the Men’s National Volleyball Team. All of the volleyball players were professional athletes of Top Volleyball League in Taiwan, and the team ranked 4^th^ in Group 3 of the 2016 FIVB Volleyball World League. They belonged to the highest level of intercollegiate athletes based on the classification of Chinese Taipei University Sports Federation in Taiwan, and they have represented Taiwan in international competitions. The non-athlete control group was recruited from Chung Yuan Christian University who had not been engaged in regular physical training of any sport.

All participants were right-handed, had normal color vision, and normal or corrected-to-normal visual acuity. They reported no history of neurological or psychiatric disorders. Before participating in the study, all participants gave written informed consent in accordance with the Declaration of Helsinki and were approved by the Research Ethics Committee of the National Taiwan University (NTU), Taiwan.

### Demographic questionnaire

Before taking cognitive tests, all participants reported their demographic and training information on a questionnaire (see S1 File). The questionnaire items include age, education, and gender, commencement age (of their sports specialty), type, total duration, number of daily and weekly practice hours, and duration of each training session. Participants were asked to answer the question based on their experience within six months recently. Measures related to the mean level of sports activity and training background were then compared among groups with one-way ANOVA (see Table 1).

**Table 1:** Demographic information of each group.

Sports Type	Badminton (F/M)	Volleyball (F/M)	Control (F/M)	*P*
**Gender**	1.34(12/23)	1.45(13/16)	1.63(17/10)	.79
**Age**	22.74(±3.4)	23.55(±2.8)	22.81(±3.2)	0.565
**Education**	15.71(±2)	15.66(±1.6)	16.37(±0.8)	0.193
**Highest Level of Competition**	3.4(±1.6)	4.9(±1.6)	N/A	< .001
**Starting Age**	10.06 (±1.4)	10.83(±1.7)		.059
**Years of Training**	11.31(±3.1)	11.57(±3.1)		.748
**Practice Sessions Per Week**	5.8(±0.58)	5.14(±0.83)		< .001
**Hours Per Session**	5.6(±0.8)	4.2 (±1.5)		< .001

Note. F = female; M = male. ± Standard deviation.

### General procedure

The participants were tested in a laboratory space. The entire experimental session took approximately 90 minutes to complete. Behavioral tests were administered in the following order for all participants: 1) Stop Signal Task; 2) Task-Switching task; 3) Partial report test; 4) change detection task; 5) the attention network task (ANT). These tasks fell into five cognitive categories: (a) inhibition control (1), (b) attentional Shifting, (c) visual spatial attention, (d) visual sensory memory (4), and (e) attentional processing (5). To mitigate potential impact of fatigue, there was a one-minute break between consecutive tests.

### Apparatus

All experiments were conducted on a PC-compatible laptop with a 14” display. Participants made responses on the keyboard. The task was displayed with custom software written in C-language (i.e., STOP-IT) [29], PsychoPy [30], or PEBL [31].

### Cognitive batteries

#### Inhibition control: Stop signal task (SST)

The SST in the current study adopted the stimuli and the procedure described in Verbruggen, Logan, and Stevens (2008). In this paradigm (See S1 Fig), participants were instructed to make speeded choice response to the shape of the cue (circle or squared), but to withhold response when a second stimulus (the stop signal) was presented. The interval between go and stop signals (Stop-Signal Delay; SSD) was adaptively adjusted to find the SSD at which participants successfully withheld responses for 50% of the stop trials on average. The first index of SST is the probability of responding on stop-signal trials, p(respond|signal), in other words, (successful stopping rate) SSR [32]. The second index of inhibitory control is an estimate of the covert latency of the stop process, stop-signal reaction time (SSRT). The third index is go reaction time (RT) on no-signal trials. RT is typically longer in blocks in which stop signals can occur than in blocks in which no signals occur. This RT difference has been interpreted as a measure of proactive control: people increase response thresholds and generally suppress motor output in situations in which stop signals can occur, compared with situations in which they can always respond [33–35]. Two core values in SST are SSD, which represents the interval between the appearance of the target stimulus and the stop-signal, and SSRT that represents the latency of the stop process, i.e. the time it takes one to complete the inhibitory process after the appearance of the stop-signal.

#### Attentional shifting: Task-Switching task (TSWT)

The TSWT assesses the participant’s ability to switch attention and response rapidly between the two sets of rules associated with identical stimulus configuration [36–38]. Details of the task paradigm are demonstrated in the S2 Fig. In brief, participants were required to determine whether an Arabic number presented at the center of the computer screen was odd or even, or it was high or low (i.e., like larger or smaller than five). The color of the background display indicated which task participants had to perform on every trial. Participants first completed two single task blocks when they only performed numerical comparison or oddity judgment, respectively, followed by two switching blocks in which oddity and number comparison occurred either every five trials or in completely random order. There were two dependent measures: the global cost indicates the difference in response time (RT) between repetition trials in the mixed task blocks and the same trial type in the single task blocks; the local cost indicates the RT difference in performance between the switch and repetition trials (in mixed-tasks blocks). More efficient ability in switching between two different tasks is expected to result in smaller costs. Moreover, in these two single task blocks (i.e. simple reaction time task), we also measured their basic processing speed since participants were asked to press when the background color present.

#### Visual-spatial attention: Change detection task (CDT)

The change detection task measures visual spatial attention and visual working memory [39]. We used a version of change detection flicker paradigm was from PEBL test battery that adopted fields of multiple circles [31,40].The procedure is demonstrated in the S3 Fig. In this task, participants searched for the difference between two alternating visual scenes consisting of disks with random color and position scattered on the display. The two visual scenes differed only with respect to a disk. The alternation was at a rate of 1.38 Hz and lasted for 30 seconds or until participants made a response. Participants were instructed to search for changes in color (e.g., circle changing color), location (e.g., changing the circle position), or additions/deletions (e.g., circle presence and absence). Once finding the change, participants pressed the “space” key on the keyboard instantly and then clicked with the mouse on the area of the field of circles where the change had occurred. The indices of performance in this task are the accuracy and the mean RT for correct trials.

#### Visual sensory memory: Iconic memory task (ICMT)

ICMT measures the capacity of sensory memory, and we adopted a variation of Sperling’s partial report task [41,42] which is implemented in the PEBL Test Battery [31]. The participant determined the identity of a target indicated by an arrow probe that was presented immediately after the target vanished. In each trial, eight uppercase letters were randomly selected from the letters ‘‘D,” ‘‘F,” ‘‘J,” and ‘‘K” and briefly presented on the display, forming a circle (radius = 3.5^o^) on a gray background (see S4 Fig). In addition, there were seven different target-to-probe stimulus onset asynchronies (SOA): 0, 116, 137, 179, 326, 621, or 1,210 ms. In all conditions, the cue remained on the screen until response. Performance sensitivity (d’) was calculated based on the hit rate (H) and false-alarm (F) rate by the formula d' = Z(H) − Z(F) (note: Z refers to z score of the normal distribution). The sensitivity index, d’, indicates the degree to which a participant was able to discriminate a true signal from noise. Mean efficiency values indicated that no speed-accuracy trade-offs occurred. Furthermore, the group differences were also investigated by calculating sensitivity (d-prime) to probes correctly identified as present in the array. The d-prime scores for each target-cue onset asynchrony and sport group are reported in S1 Table. They were derived by subtracting the normalized (z-score) proportion of “false alarms” from the normalized proportion of “correct hits” for each trial type. The advantage of this comparison is that it takes into account any response biases and instead tests participants’ sensitivity to the presence of a particular stimulus, relative to chance. We then compared d-prime values across each SOA were submitted to one-way ANOVA with the between-subject factor of group.

#### Attentional processing: Attention networks task (ANT)

ANT combined the flanker task and Posner’s spatial cueing paradigm to assess the efficiency of the alerting, orienting, and conflict resolution of attentional process within a single task [43]. Performance was measured by subtracting mean RTs between conditions with different combinations of cue or target types. As illustrated in S5 Fig, there were three cue conditions (no cue, center cue, spatial cue) and two target conditions (congruent and incongruent). The stimuli consisted of a row of five arrowheads pointing leftward or rightward on the gray background. The target was the central arrowhead, and the symmetrically flanking arrowheads were pointing either in the same direction as (congruent condition) or the opposite direction (incongruent condition) from the target. Participants were instructed to discriminate the direction of the central target arrow. The no-cue condition severed as the baseline, whereas the central cue served as an “alert” where the participant can be prepared for the appearance but not the location of the target. The spatial cue could have directed the participants’ spatial attention toward the target location and thus speeded up response.

### Data analysis

We compared the cognitive performance of badminton players, volleyball players, and non-athletes by analyzing the mean accuracies and RTs for the five type of neuropsychological testing. Effect sizes for each ANOVA were estimated using partial eta squared (*η*_*p*_^*2*^). Post hoc comparisons were conducted employing the Fisher’s least significant difference (LSD) correction for multiple testing. Mean RT and accuracy data were subjected to between-subject analysis of variance (ANOVA) using IBM SPSS Statistics (Version 24. IBM Corp.). The control and athletes were matched for age and years of education. For each task, the behavioral measures that best represent the cognitive constructs relevant to the present investigation were selected for the ANOVAs. Table 2 reported results of all measures.

**Table 2:** Results of cognitive battery by group.

		Badminton	Volleyball	Control
**Stop Signal**	SSR*	43.4(±6.2)	50.0(±11.5)^B,C^	45.9(±10.1)
	SSRT	300(±53)	289(±58)	274(±59)
	NSRT	736(±196)	683(±210)	643(±199)
	NSACC	97.2(±3.61)	97.1(±5.71)	96.4(±5.27)
**Task Switching**	Single RT(s)*	0.51(±.08)	0.56(±.07)	0.55(±.09)
	Global Cost**	.016(±0.024)^C^	.012(±0.023)^C^	.011(±0.1)
	Local Cost**	-.033(±0.49)^C^	-.025(±0.046)^C^	.054(±0.09)
**Change Detection**	Accuracy	.92(±.11)	.92(±.15)	.90(±.11)
	RT(s)	25.4(±10)	25.9(±14)	24.0(±8)
**Iconic Memory**	Accuracy**	.54(±0.77)^C^	.59(±0.99)^C^	.49(±.10)
	RT	1.50(±0.30)	2.16(±2.7)	1.31(±0.4)
**Attention network task**	Alert**	.002(±.04)	.028(±.03)^B,C^	-0.005 (±0.034)
	Orient	.038(±.03)	.018 (±.04)	0.032(±0.02)
	Conflict	.110(±.04)	.12(±.03)	0.12(±0.06)

**Note.** B, V, C, indicate the denoted value is significantly different from that of the badminton, volleyball, or control group in the same row. Asterisks indicate significant F-values (* *p* < .05; **p < .001) SSR = successful stopping rate; RT = reaction time; SSRT = stop signal reaction time; NSRT = no-signal reaction time; NSACC = accuracy of no-stop signal trials.

## Results and discussion

### Demographic questionnaire

Table 1 listed mean and SD of the demographic information by groups. For the two athlete groups, the results showed significant difference in the highest level of competition participation (F(1,62) = 12.51, p = .001, $\eta_{p}^{2}$ = .168; volleyball players [4.9] higher than badminton [3.4]), average practice times per week (F(1,62) = 13.88, p = .000, $\eta_{p}^{2}$ = .183; badminton [5.8] more than volleyball [5.14]), and hours spent per training session (F(1,62) = 18.96, p = .000, $\eta_{p}^{2}$ = .234; badminton [5.6 hours] more than volleyball [4.2 hrs]), whereas other demographic variables showed no significant difference (starting age of training, F(1,62) = 3.69, p = .059, $\eta_{p}^{2}$ = .056; years of training, F(1,62) = .104, p = .748, $\eta_{p}^{2}$ = .002). For variables that are applicable to all of the three groups, none reached significance, including age (F(2,90) = 0.575, p = 0.565, $\eta_{p}^{2}$ = .013), years of education (F(2,90) = 1.67, p = .193, $\eta_{p}^{2}$ = .037), gender ratio (χ^2^(2) = 5.074, p = .079). This difference represents the characteristics of sports type, indicating training modalities would differ depends on the type of sports.

### SST

No significant differences was found for stop signal reaction time (SSRT) (*F*(2,90) = 1.572, *p* = 0.213, $\eta_{p}^{2}$ = 0.035). However, the estimated successful stopping rate (SSR) was significantly different among groups (*F*(2,90) = 3.981, *p* = 0.022, $\eta_{p}^{2}$ = 0.083). Post hoc comparison showed higher SSR in the volleyball players (50%) than the badminton players (43.4%, *p* = 0.006) but not the control (45.9%, *p* = 0.110).

### TSWT

Averaged reaction times on the global (*F*(2,90) = 29.51, *p* = 0.0001, $\eta_{p}^{2}$ = 0.401) and local cost (*F*(2,90) = 14.87, *p* = 0.0001, $\eta_{p}^{2}$ = 0.253) showed significant between group differences. Post hoc comparisons revealed that both types of athletes have greater ability to switch between two different tasks than control groups (p = .039). Moreover, we also observed significant group difference on single block of task-switching paradigm (*F*(2,90) = 4.177, *p* = 0.019, $\eta_{p}^{2}$ = 0.09). Post hoc comparison showed badminton players have better performance than volleyball players (*p* = 0.009) and controls (*p* = 0.036).

### CDT

No significant group difference was found in the RT of change detection (*F*(2,90) = 0.051, *p* = 0.95, $\eta_{p}^{2}$ = 0.005).

### ICMT

No group difference was found in RT of the ICMT (*F*(2,90) = 2.265, *p* = 0.110, $\eta_{p}^{2}$ = 0.0049). The ANOVA on accuracy showed significant differences among groups (*F*(2,90) = 7.52, *p* = 0.001, $\eta_{p}^{2}$ = 0.146). Post hoc comparisons showed that volleyball players (p < 0.001) and badminton players (p = 0.049) have higher iconic memory recall rates than control group.

**Fig 1:**
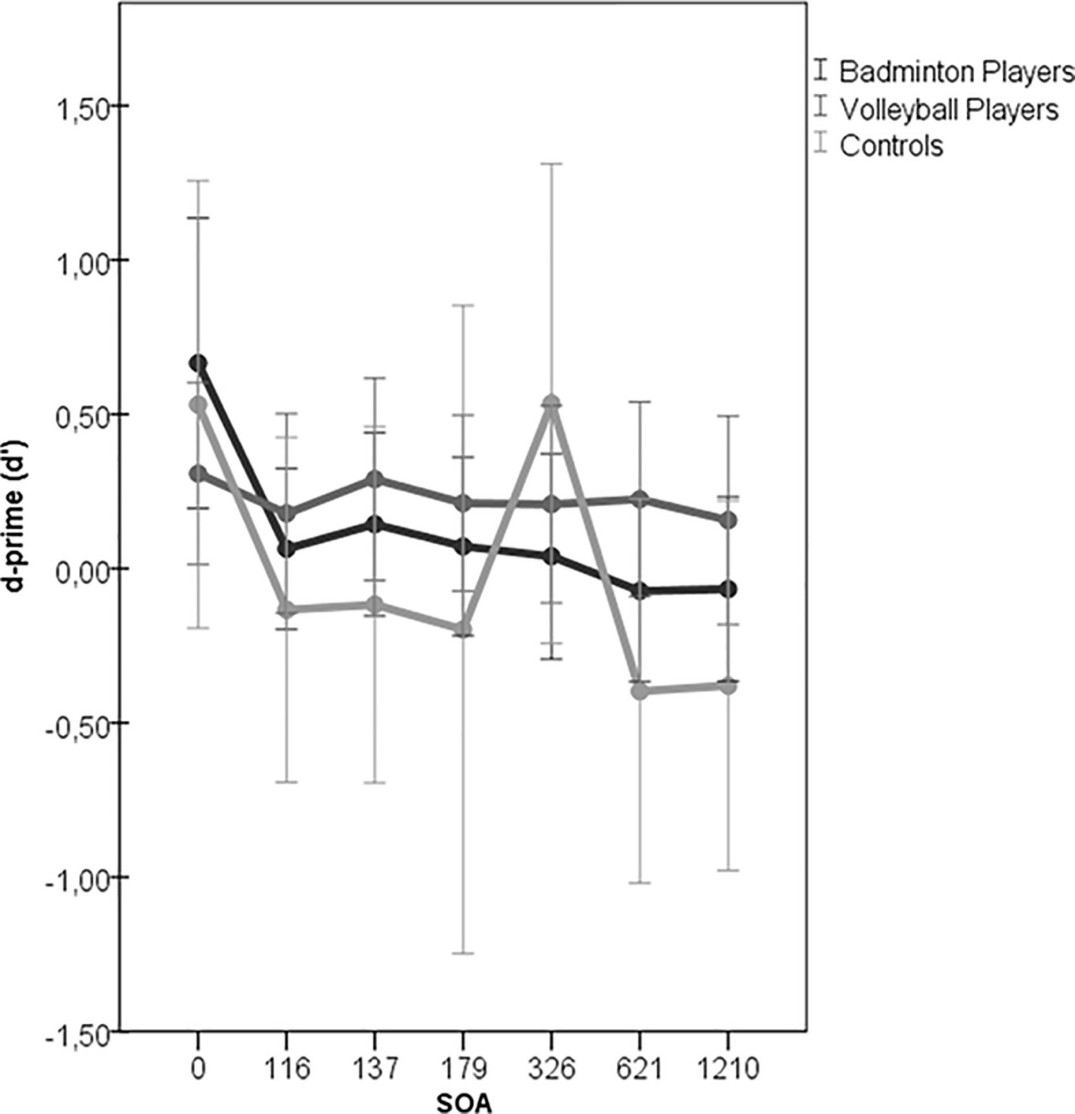
Mean d-prime of each group across all SOAs. * indicate a significant difference between groups p<0.05; ** = p<0.001. Error bars represent ±1 standard deviation. SOA = stimulus onset asynchrony.

D-primes were also estimated for trials correctly identified (See S3 Fig for each target-cue onset asynchrony and sports group), and were subjected to a two-way mixed design ANOVA (Group × SOA; Fig 1). Overall, the athlete groups had significantly better performance than the control group (*p* < 0.05) at the SOAs of 137ms, 326ms, 621 ms and 1210 ms. In addition, volleyball players also showed slower decay rate than badminton players (p = 0.039) on iconic memory. Badminton players appeared to have better visual persistence which can be seen from results at 0 ms condition with higher accuracy. The results of other groups appear less stable. Performance at different SOAs were highly variable and inconsistent with those reported in previous literature, which could be attributed to the total number of trials and time limit to test all the cognitive task.

Regarding the performance across different SOAs, the decay function demonstrated that athletes generally have higher iconic memory capacity compared to control, indicating better ability to retain impressions of sensory information at a glance. In contrast, control group showed lower iconic memory capacity, and individual performances across different SOAs varied a lot more than athlete groups (mean variance across SOAs in badminton = ±0.32, volleyball = ±0.31, and control = ± 0.7).

### ANT

Alerting score of attentional network task (*F*(3,63) = 7.09, *p =* 0.001, $\eta_{p}^{2}$ = 0.141) showed significant main effect of between group differences. Specifically, volleyball players showed better performance on alerting states of attention network score (.028) than badminton players (.002; p = .005) and controls group (-.005; p = .001). Comparison between badminton player and control group found a certain trend toward significance (p = .053). Orienting and conflicting networks of attention did not show any significant results between groups (*F*(2,90) = 2.339, *p* = 0.102, $\eta_{p}^{2}$ = 0.051 and *F*(2,90) = 0.516, *p* = 0.599, $\eta_{p}^{2}$ = 0.012, respectively).

## Discussion

The aim of the present study was to examine how different sports expertise may be associated with measures of different aspects of cognitive functions. Specifically, we aim to explore the relationship between strategic sports (volleyball players) and interceptive sports (badminton players) on cognition in general. As expected, athletes outperformed ordinary people in many aspects: we found that both athlete groups did have superior performance on visual sensory memory (partial report paradigm) and attentional shifting (task switching paradigm). Specifically, both volleyball and badminton players showed longer decay rate of sensory recall and lower transfer cost between two different tasks. Further, volleyball player not only have better performance than control group on such cognitive abilities but also outperformed badminton players on ability to better usage of temporal information to prepare for upcoming stimuli in alerting states of attention and to inhibit proponent motor response. These evidence suggesting volleyball players have boarder cognitive skills that require stimuli-driven visual attention and inhibitory control of action. Moreover, badminton players showed faster reaction time from the performance of simple reaction time in badminton players’ trials in the single task block of task switching, indicating that training in interceptive sports may be related to superior performance on basic sensorimotor speed. Overall, volleyball player (i.e. strategic sport) demonstrates superior performance on stimuli-driven visual temporal attention processing and motor inhibition, while intercept sport show faster sensorimotor speed.

The current results are consistent with previous studies suggesting that athletes have better performance on processing speed (see S6 Fig) [9,14,16,44]. For example, two studies have reported that superior performance on sensorimotor speed was observed in tennis players and soccer players [9,16].Recently, a study conducted a series of perceptual speed tests on professional baseball players and found not only higher processing speed in athletes, but also applied the perceptual-cognitive measures to predict their actual field performance [45]. These results indicate interceptive sport may have a superior motor speed that is subject to training modalities. However, lacking a direct comparison between different types of sports under the same experimental setting may undermine the explanation. Hence, the current study adds to this line of findings with a direct comparison between interceptive sports (i.e., badminton) and strategic sports (i.e., volleyball), showing faster sensorimotor speed in interceptive sports are possibly subject to different training modalities. Nonetheless, the findings may explain with cautious since motor abilities are genetically inherited that are prerequisites for performing various sports skills. Future studies may want to consider individual genetic variations whether genetic makeups and the extensive amount of training contribute to athletic superiority of motor speed.

However, unlike previous studies showing faster change detection reaction time in the athletes than the non-athletes control [38], we did not replicate this group difference in the change detection paradigm (CDT). As change detection might generally require memory-guided spatial attention, it may be less related to sports experience. Unlike other sports, volleyball and badminton player are quite similar in terms of courts size and both need two sides of court divided by net. During the match, both are only one serve attempt allowed and exchange of consecutive hits. As such, both sports rely on more visually guided spatial attention and may have shorter spatial attention span since similar court size and less dynamic playing environment than other team sports. Another possible reason is that the current stimuli configuration of the change detection task resemble real-world scenario to a greater extent than previous studies. Future studies may find it worthwhile to manipulate different types of stimuli (e.g., shape or object) embedded in the sport-related scenes and examine how sensitivity to changes in these more naturalistic scenes differs across groups of elite athletes.

Also at odds with previous reports [8], badminton players here showed lower rate of successful stop in the stop signal paradigm than both the volleyball players and the non-athlete control. Badminton players may not involve as much action inhibition during the match as we expected. Compared to team sports, badminton sports are considered as one of the fastest sports, which require speedy reflexes but not so much the ability to withhold their intention to react.

Our results also demonstrate that volleyball players outperforms badminton players on task switching, in which the former had smaller costs both between different task and within task repetitions, indicating that volleyball players may have a greater cognitive flexibility to shift between two different tasks. Optimal performance in competitive sport involves the efficient operations of a variety of cognitive functions, especially so for competitive team sports. Elite team sports athletes have to put together processing of a substantial amount of information about performing tactically, deceptions in action, time-limited decision making, and interact directly with teammates and opponents under complex behavior and dynamic environments. All of these aspects may conjointly explain why team sport experts generally outperform badminton players.

Moreover, some may concerned whether gender difference existed in these sports expertise and manifested in these cognitive functions. Previous study already showed that sport expertise would minimize gender differences [15], if they occurred, would only be observed in the control group, not in the athlete group. The reason behind this hypothesis is that systematic sport training and practice appear to reduce gender differences in spatial ability. In this sense, it might be the case that gender differences within a sport on tasks involving perceptual-motor speed are minimized if male and female athletes are given equal opportunities for similar experiences, learning, and training [46]. This idea would explain why the gender differences, when they occurred, were only present in the control group in our study, not in the athlete group. In this study, as hypothesized from previous studies, we sought to test if gender difference exists among those cognitive measures. We found no difference across all group on all cognitive measures except for SSRT (P = 0.026). We further test if this effect exist when we compare this index in each group separately, result showed no significant difference was seen in this measure (p = 0.213).

Though the current study observed several lines of evidence regarding effects of sports specialty on cognition, some limitations need to be considered. For example, although we have recruited quite a few numbers of athletes to participate, the sample size is still limited. Because the nature of their training and outstanding sports expertise, professional athletes in this study are quite difficult to access and recruit, and hence the sample size is unavoidably small. Furthermore, the cycle of training differ between types of spots expertise may also add to the confounding in results. For instance, some participants were preparing for major competition in months, whereas others just finished their competition in days. This may increase the variance of performance among types of sports and individual differences. Moreover, Voss et al. (2010) suggested that future sport-cognition studies should match appropriately gender variable and use a diverse range of sports types and levels of expertise. They argue that sports types would be a potential moderator variable that exerts characteristically different sets of mental demands and distinct experience-dependent plastic changes in athletes. Future studies would benefit from recruiting professionals in sports science to overcome some setbacks mention above.

In addition, there has been accumulating evidence supporting that level of physical activity and cardiovascular fitness have a positive effect on cognition across the life span [2–4]. It has been suggested that increasing physical activity and fitness levels may facilitate academic performance in children [47,48] and enhance daily cognitive functioning in the preservation and alleviate age-related cognitive decline in the healthy elderly [1]. Nonetheless, although substantial evidence has reported a beneficial effect on various aspect of cognitive function after physical exercise, yet the cognitive mechanism of long-term physical training in highly skilled individual on the functional brain are still unclear. On possible mechanism is that fitness level are key to improve cognition and brain health (e.g., [49–52]). One more limitation of the current study is that we did not monitoring their physical fitness level in these professional athletes and controls. Future study should consider the use of objective ways, such as monitoring their heart rate or recording physiology index, to estimate the athlete’s fitness. An additional potential confound would also suggested for future studies is anthropometric variables, which would able to further the knowledge on the influence of weight, height, and body mass index.

## Conclusions

To conclude, the results presented here demonstrated expertise effects which substantiate the view that laboratory tests of cognition may indeed enlighten capture the sport-cognition relationship, even though only supported by only a limited number of measures and tasks have been tested in the present study. The results suggest that the effects of sport expertise on visual sensory memory, attention shifting, basic processing, and alerting of attention network are reflected essentially in measures of response time and accuracy, in attention shift, alerting attention and simple reaction time tasks, which is in accordance with the specific cognitive demands of interceptive sports and also consistent with previous findings (e.g., Voss et al., 2010). Our results also partially echoed previous studies on the superiority of volleyball players on the selective task of cognitive battery.

Nevertheless, there is still much to be learned about cognitive-perceptual expertise in sports. Overall, volleyball player has better performance on visual sensory memory, attention shift, stopping the behavior, and better usage of temporal information at alerting state of attention. Longitudinal studies tracking athletes along various levels would be highly informative for understanding how cognitive abilities differ as a function of a priori broad cognitive abilities, experience (years of training), and types of training. Ultimately, the study of cognitive-perceptual expertise in sport has great potential to assist trainers in the cultivating future elite athletes and to provide insights into how brain function differs following different sports experience.

## Supporting information

S1 Fig Schematic illustration of the stop signal paradigm. (TIF)

S2 Fig Schematic illustration of the task switching paradigm. (TIF)

S3 Fig Schematic illustration of the change detection paradigm. (TIF)

S4 Fig Schematic illustration of the partial report paradigm. (TIF)

S5 Fig Schematic illustration of the attention network task. (TIF)

S6 Fig Average reaction time of single task blocks (simple choice reaction time task). (TIF)

S1 File Demographic questionnaire used in this study. *Note the original version of this questionnaire is in Traditional Chinese, direct translation into English by using Google Translate.
(PDF)

S1 Table The d-prime scores for each target-cue onset asynchrony across sport groups. (TIF)
