# Supplementary figures and images for: Team sport expertise shows superior stimulus-driven visual attention and motor inhibition
Source: PLoS One. 2019 May 15;14(5):e0217056. doi: 10.1371/journal.pone.0217056 (PMC6519903; doi:10.1371/journal.pone.0217056)

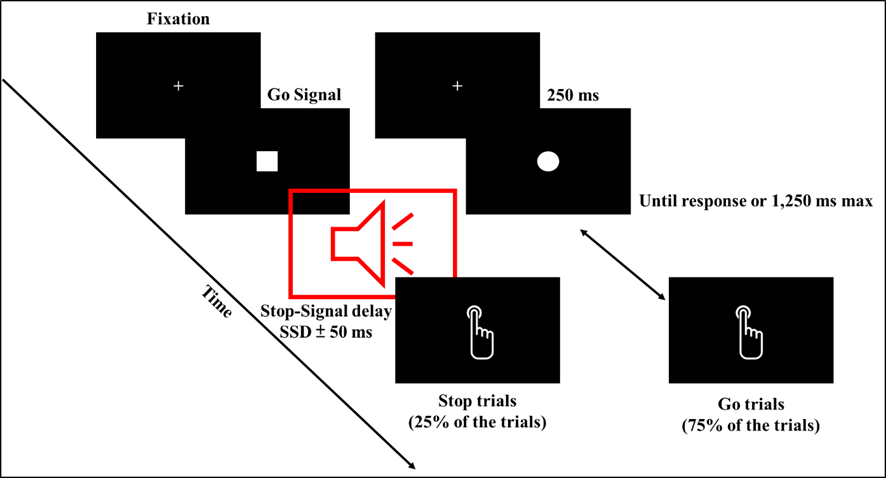

Supplement: S1 Fig — (TIF) [file pone.0217056.s001.tif]

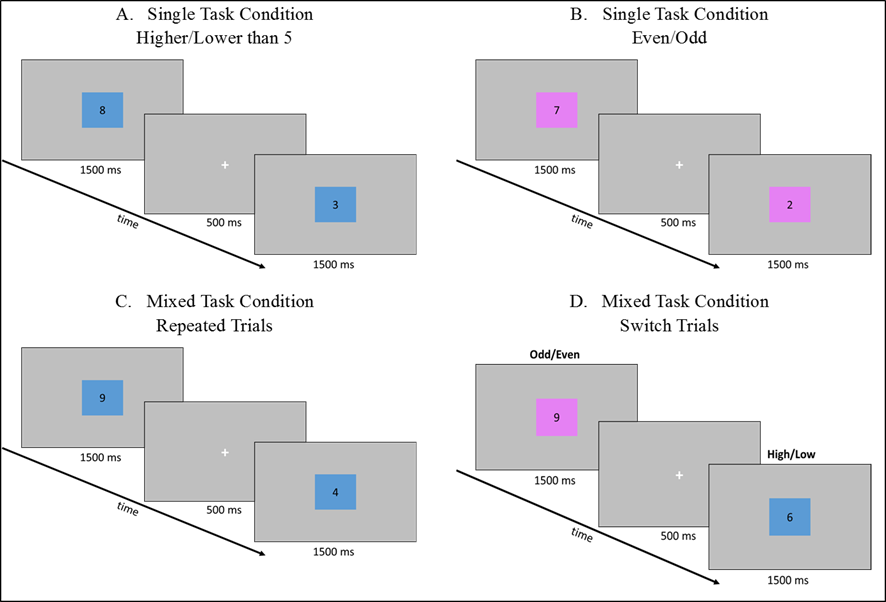

Supplement: S2 Fig — (TIF) [file pone.0217056.s002.tif]

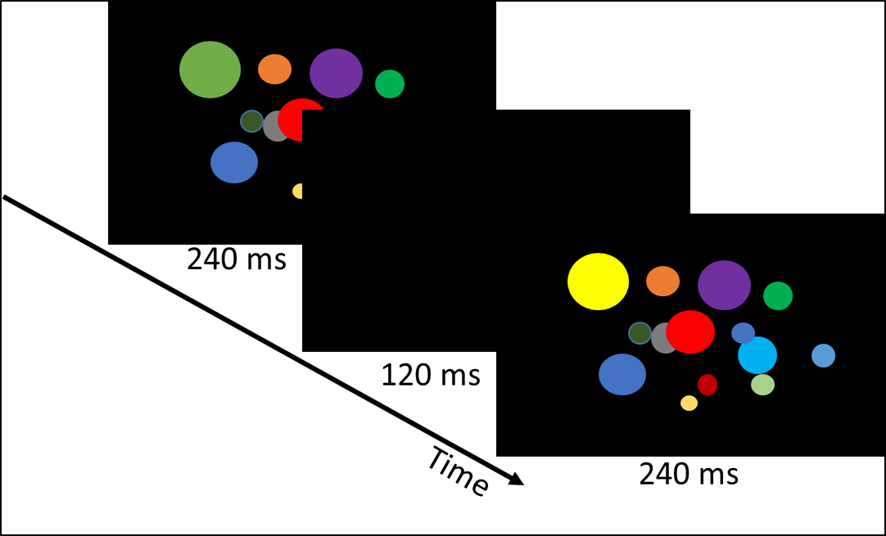

Supplement: S3 Fig — (TIF) [file pone.0217056.s003.tif]

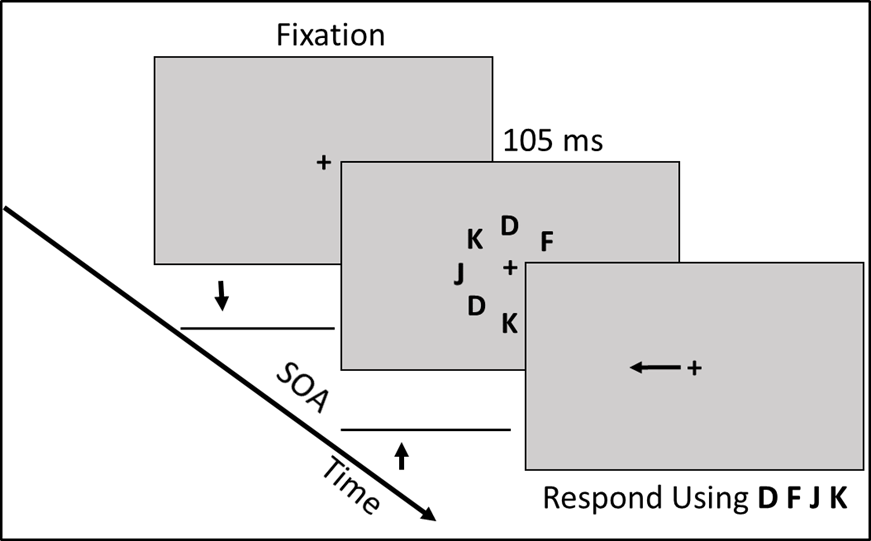

Supplement: S4 Fig — (TIF) [file pone.0217056.s004.tif]

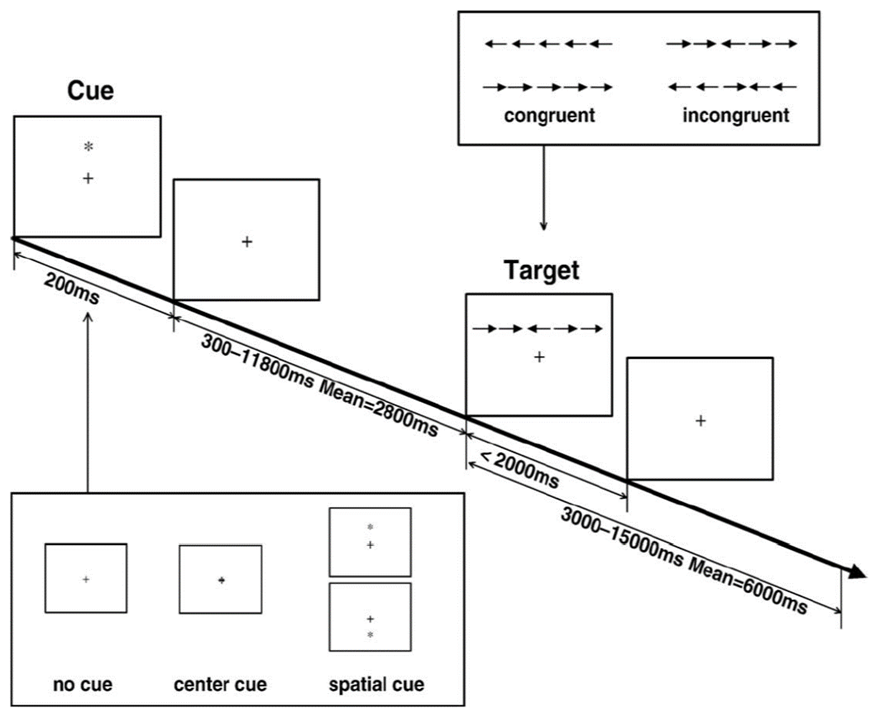

Supplement: S5 Fig — (TIF) [file pone.0217056.s005.tif]

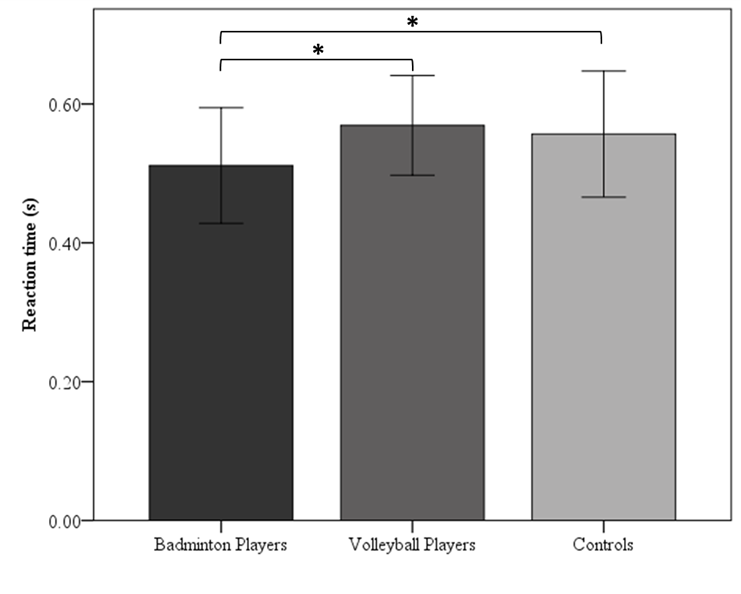

Supplement: S6 Fig — (TIF) [file pone.0217056.s006.tif]

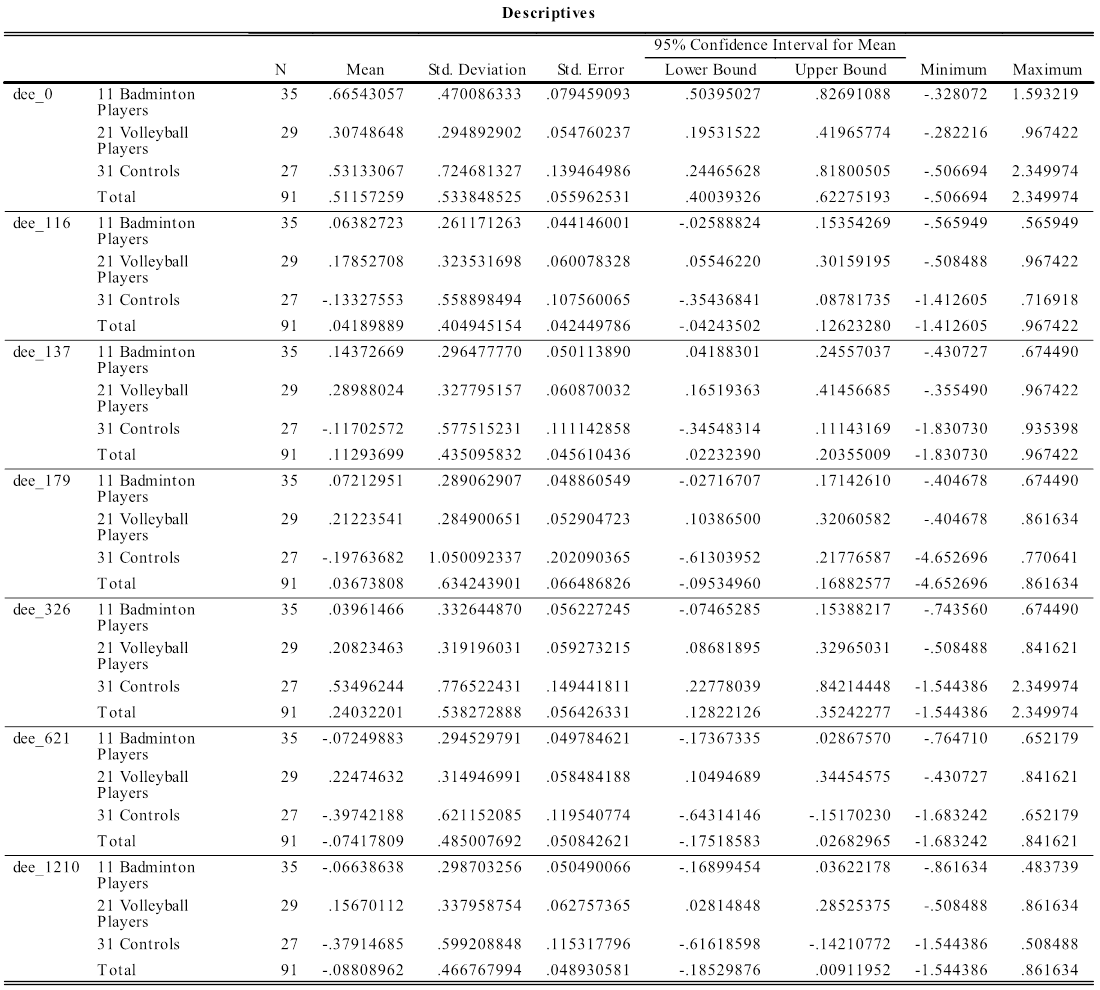

Supplement: S1 Table — (TIF) [file pone.0217056.s008.tif]
