# Supplementary material for: Team sport expertise shows superior stimulus-driven visual attention and motor inhibition
Source: PLoS One. 2019 May 15;14(5):e0217056. doi: 10.1371/journal.pone.0217056 (PMC6519903; doi:10.1371/journal.pone.0217056)
Supplement: S1 File — *Note the original version of this questionnaire is in Traditional Chinese, direct translation into English by using Google Translate. (PDF) [file pone.0217056.s007.pdf]

Basic information of experimental participants

The research plan host will treat any document that identifies your identity and your personal privacy as confidential, and will never be disclosed. Your identity will be sufficient and confidential when publishing research results in the future. Participants can be directly reviewed by monitors, auditors, research ethics committees, and competent authorities to ensure research processes and data, in compliance with relevant laws and various regulatory requirements; these individuals are committed to maintaining the confidentiality of your identity.

* Required

Experiment Participant Number *

Please answer by the experimenter.

Your answer

Name *

Your answer

Contact number (mobile number) *

Your answer

Gender *

- [ ] male
- [ ] female
- [ ] Other:

Date of birth (Eastern) *

Date

mm/dd/yyyy

Highest education or education *

- [ ] National small
- [ ] secondary
- [ ] High school
- [ ] Five-year college
- [ ] Two-year technical college
- [ ] the University
- [ ] graduate School
- [ ] Other:

Habitual hand *

- ○ Left hand
- ○ Right hand

Special sports background data survey

Please answer according to your current training status.

Have you participated in those sports events (domestic and foreign)? *

- [ ] National Secondary School Games
- [ ] National college sports meeting
- [ ] National Games
- [ ] National Games
- [ ] World University Games
- [ ] Asian Games
- [ ] Olympia Games
- [ ] Other:

How do you start regular special sports training from a few years old? *

Your answer

What is your special sports training for several years? *

Your answer

How many days do you train for a week? *

- Two days
- Three days
- Four days
- Five days
- Six days
- Seven days
- Other:

Is the training time about a few hours a day? *

- 1 hour
- 2 hours
- 3 hours
- 4 hours
- 5 hours
- 6 hours
- Other:
